# Numerical emulation of Thru-Reflection-Line calibration for the de-embedding of Surface Acoustic Wave devices

**DOI:** 10.1038/s41598-018-27511-0

**Published:** 2018-06-18

**Authors:** D. Mencarelli, B. Djafari-Rouhani, Y. Pennec, A. Pitanti, S. Zanotto, M. Stocchi, L. Pierantoni

**Affiliations:** 1 Università politecnica delle Marche, Ancona, Italy; 2 Institut d’Electronique de Microelectronique et de Nanotechnologie, University of Lille, Lille, France; 3 Consiglio Nazionale delle Ricerche, NEST Lab., Pisa, Italy; 4 National Institute of Nuclear Physics, Frascati, Rome Italy

## Abstract

In this contribution, a rigorous numerical calibration is proposed to characterize the excitation of propagating mechanical waves by interdigitated transducers (IDTs). The transition from IDT terminals to phonon waveguides is modeled by means of a general circuit representation that makes use of Scattering Matrix (SM) formalism. In particular, the three-step calibration approach called the Thru-Reflection-Line (TRL), that is a well-established technique in microwave engineering, has been successfully applied to emulate typical experimental conditions. The proposed procedure is suitable for the synthesis/optimization of surface-acoustic-wave (SAW) based devices: the TRL calibration allows to extract/de-embed the acoustic component, namely resonator or filter, from the outer IDT structure, regardless of complexity and size of the letter. We report, as a result, the hybrid scattering parameters of the IDT transition to a mechanical waveguide formed by a phononic crystal patterned on a piezoelectric AlN membrane, where the effect of a discontinuity from periodic to uniform mechanical waveguide is also characterized. In addition, to ensure the correctness of our numerical calculations, the proposed method has been validated by independent calculations.

## Introduction

Surface acoustic waves (SAWs) are object of intense study, owing to the increasing ability to manufacture thin film structure and to the variety of possible applications^1–4^. The use SAW devices as electromechanical filters^5–7^ for frequency control and selection, based on piezoelectric characteristics of a substrate or layer, is well known from decades: an evaluation of the morphology, implementation, and potential of SAW filters in communication devices, in terms of size, weight, cost, losses, bandwidth, and overall performance, can be found, for example, in ref.^8^. As several papers^9–14^ pointed out, SAW-based devices have high potential for sensing applications. The interest on SAW sensors include not only gravimetric applications, but also bio-applications - owing to chemical and biochemical adsorption taking places at surfaces in liquid environments -, by means of delay-line or resonating configurations, even in combination with microfluidic systems.

High-performance interdigitated transducers (IDTs), made of metal fingers deposited on piezoelectric substrate, provide an effective way to excite SAWs. Modelling of IDT can be carried out by means of circuit models^15,16^ and “hybrid” - electrical and mechanical - impedance representations^17^. Similar analysis is done in refs^18–22^, by means of lumped circuit models of the transduction. A review of standards methods for the analysis of IDTs, including Coupled Mode Theory, can be found in^23,24^. All these models relies on a proper parametrization of the underlying physics and of propagation/reflection effects. However, parametrization is not generally easy to achieve, and difficulties may arise in fine description of general or arbitrarily shaped IDT geometries, abrupt discontinuities and boundary finite terminations, real electrode shape, surface roughness, multilayer complex structures, multimodal conditions with many propagating/attenuating surface waves, rigorous diffraction analysis of the coupling to substrate modes.

The above issues can be rigorously addressed by means of multihysic simulators based on finite-element methods^25^ (FEM), with the possible cost of heavy meshing and high computer time/memory. However, it should be remarked that presently available simulators can provide accurate and fast results even in case of complex computational domains, as they usually feature highly effective sub-griding, iterative/adaptive mesh refinement, smart domain decomposition, and iterative numerical solving tools.

In this work we provide a direct procedure to characterize mechanical excitation by IDT, based on Scattering-Matrix (SM) representation, and on Thru-Reflection-Line (TRL) calibration, which has never been reported so far, up to our knowledge.

From the modelling point of view, FEM has been applied to electrical loading due to patterned IDT electrodes over piezo-substrates. The importance of the procedure presented here resides in that it allows to isolate and de-embed the acoustic components of SAW-based devices, such as opto-mechanical (OM) cavities, filters, phononic crystals/metamaterials, nonlinear and/or active elements, from the outer IDT structure, potentially large and bulky. In this regard, TRL calibration can be of help, not only in the analysis, but alsoin the design & synthesis of the above devices.

## Results

### Simulation strategy

**Figure 1:**
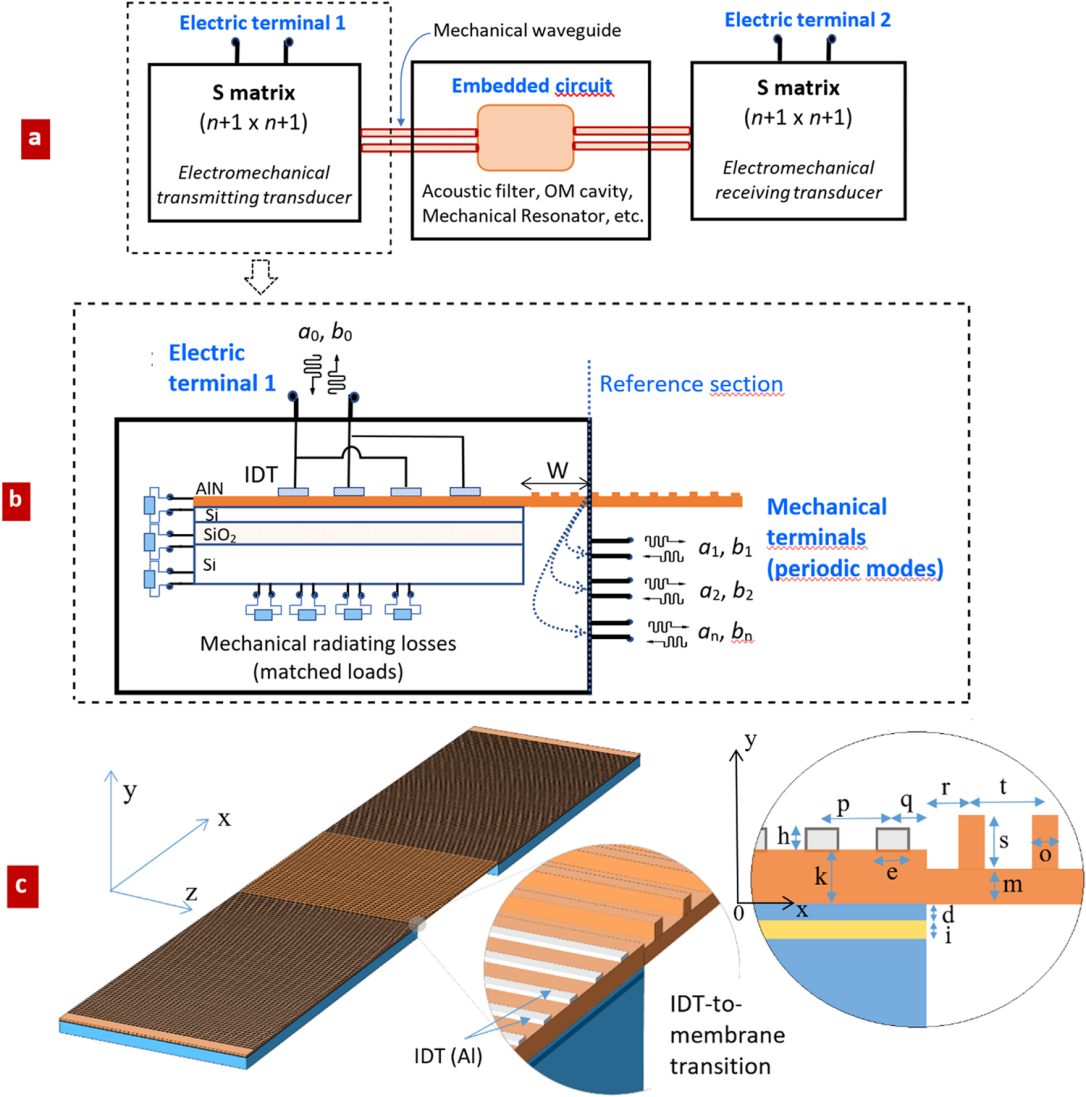
Scheme of the simulated SAW-based structure; (**a**) two port circuit, with two electromagnetic ports, plus *n* mechanical internal ports; (**b**) detail of the transition from IDT to periodic mechanical waveguide; (**c**) simulated and structure, with transitions from IDT fingers to periodically patterned mechanical waveguide (central suspended membrane); geometric parameters: *h* = 0.2 µm; *k* = 1 µm; *p* = 2.35 µm; *q* = p/2; W = 16*p*; *e* = 0.65 µm; *t* = 2.65 µm; *r* = t/2; *s* = 0.75 µm; *d* = 0.22 µm; *i* = 0.24 µm; *m* = 0.77 µm; *o* = 0.72 µm.

In this contribution, the transition from electromagnetic source to mechanical waves, propagating in periodic crystal structures, is rigorously simulated by means of a general circuit representation, that makes use of the SM formalism. Forward and backward waves are defined at the transmitting/receiving electrical terminals (IDTs) of a SAW-based device, as shown in Fig. 1. The corresponding global (2 × 2) S-matrix includes the effects of three cascaded blocks (Fig. 1a): (i) the left transition IDT-to-mechanical waveguide, (ii) an internal SAW component, e.g. an acoustic resonator, and iii) the right transition from mechanical waveguide-to-IDT. The first and third blocks can be characterized by using a number *n* + 1 of ports, where one external port is the electric terminal and the other *n*, defined as internal ports and depending on the size of their waveguide, model the mechanical excited waves. Another set of ports can be virtually associated to the mechanical modes radiated into the substrate and its surrounding regions (Fig. 1b), but since they just are terminated to their matched loads, they’re accounted in the simulation by the means of the perfect matched layers (PML).

The actual device considered for simulation is shown in Fig. 1c: to have an effective electromechanical action, we assume a large IDT (≈176 µm long), formed by 75 metal fingers, with alternating voltage in quasi-static conditions. All geometric parameters are reported in the legend of Fig. 1 (that for the sake of clearness, does not maintain the scale in the picture): in particular, the spatial period (unit cell along the *y*-axis) of the IDT, and of the subsequent mechanical waveguide, are respectively *p* = 2.35 µm and *t* = 2.65 µm. The IDT parameters have been numerically optimized to have a high mechanical coupling to the membrane.

### Multiphysic analysis of surface acoustic waves in uniform waveguides excited by IDT

In this section, we shortly recall the main concepts involved in a multiphysic simulation where the equations of the electromagnetic and mechanical physics are coupled. With reference to the structure shown in Fig. 1, the electrical potential *V* (defined in the whole computational domain) is connected self-consistently to the SAW propagating in a piezoelectric AlN layer placed on a multilayer made of Si/SiO_2_ materials, with the IDT placed on the top. The actual multilayer composition is not relevant for the scope of the present work, where the objective is to consider a realistic and complex practical situation to verify consistency of the simulation as well as the robustness of the numerical analysis. Due to the electromagnetic field large wavelength with respect to the size of the device under study and to the mechanical wavelength, a quasi-static approximation is assumed.

The general formulation for the mechanical displacement **U** (*u*_*i*_*, i* = *1*, *2*, *3*) along the axis (*x*, *y*, *z*) of the Cartesian coordinate system depicted in Fig. 1c, and the corresponding stress tensor **T**, leads to a system of partial differential equations combined with a system of piezoelectric constitutive equations: the stress tensor **T**, the electric field **E**, the electric displacement **D** and the strain tensor **S** are all coupled together. In particular, the link between (**T**, **D**) and (**E**, **S**), not reported here for brevity, is provided by the matrices of elastic stiffness tensor **c**, the piezoelectric tensor **e**, and the dielectric permittivity tensor^6^ **ε**. The equation of motion for a generic piezoelectric material of density *ρ* is expressed by:

1 $$\rho \frac{{\partial }^{2}{u}_{i}}{\partial {t}^{2}}=\sum _{j}\sum _{k}({e}_{kij}\frac{{\partial }^{2}V}{\partial {x}_{j}\partial {x}_{k}}+{c}_{ijkl}^{E}\frac{{\partial }^{2}{u}_{k}}{\partial {x}_{j}\partial {x}_{l}})$$

in combination with the constrain div **D** = 0, coming from the assumption of insulating material:

2 $$\sum _{i}\sum _{j}({\varepsilon }_{ij}^{S}\frac{{\partial }^{2}V}{\partial {x}_{i}\partial {x}_{j}}-\sum _{k}{e}_{ijk}\frac{{\partial }^{2}{u}_{j}}{\partial {x}_{i}\partial {x}_{k}})=0$$

In what follows, the SAW device will be considered large in the *z* direction, and a 2D analysis will be performed. The displacement components in the (*x*, *y*) plane will be referred to as (*u*, *v*).

**Figure 2:**
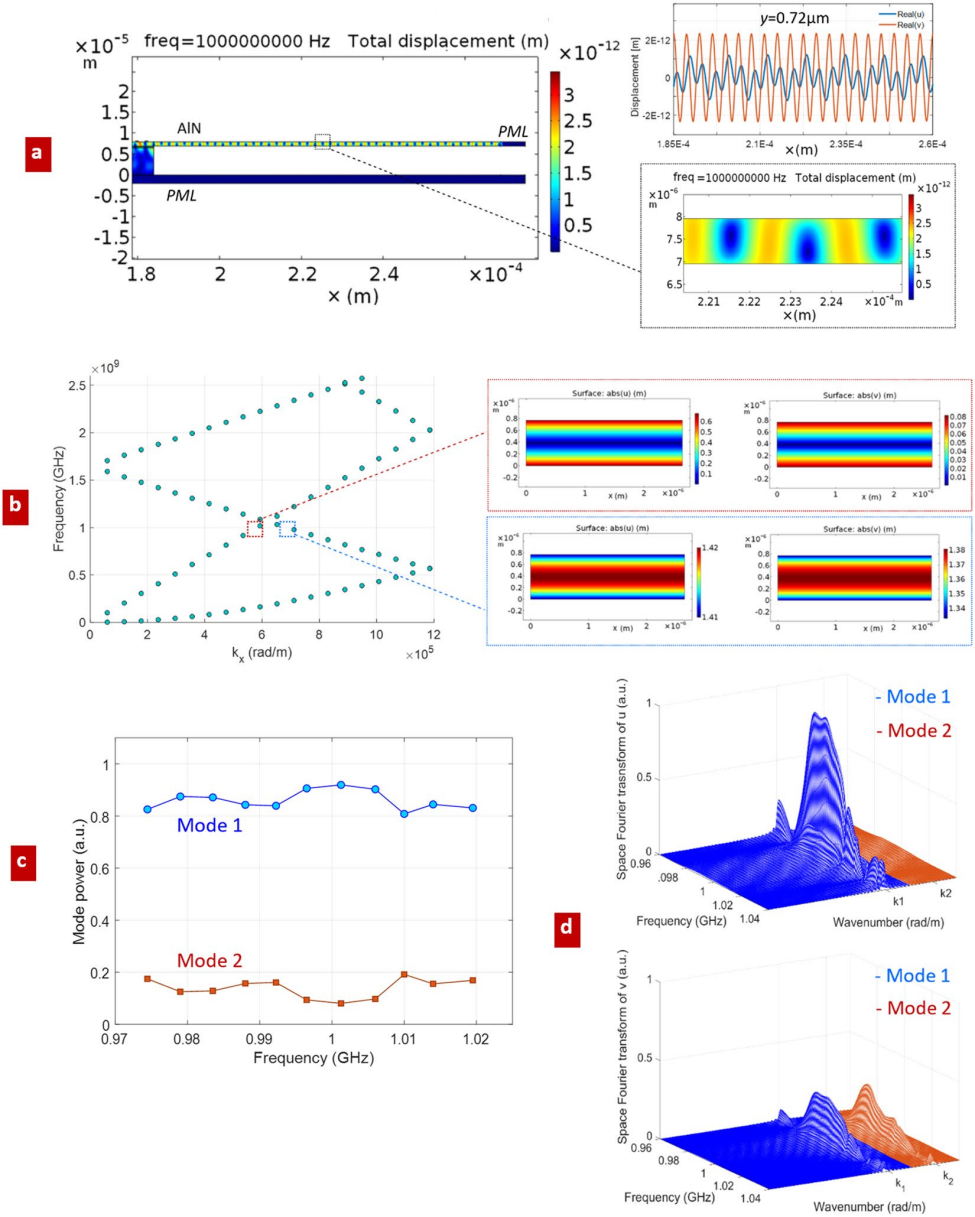
Simulation of mechanical waves excited in the suspended uniform AlN membrane; (**a**) calculated displacement, i.e. $d=\sqrt{{|u|}^{2}+{|v|}^{2}}$, and (*u*, *v*) profiles along a cut line in *x* direction, at fixed *y* = 0.72 µm; (**b**) dispersion curves of the membrane and its two modal solutions @ 1 GHz; (**c**) relative powers of the excited mechanical modes; (**d**) space Fourier transform of *u* and *v* sampled along the same cut line as in (**a**), vs frequency.

For the case of a uniform AlN suspended membrane of thickness 1 µm, excited by the 75 finger IDT of Fig. 1c, typical displacement results are reported in Fig. 2a. In such analysis, the physical quantities are expressed by means of electromagnetic variables, namely the voltage potential *V* and the driving current *I* at the input terminals of the IDT, and mechanical variables, i.e. stress forces and surface velocities. In the S-matrix representation, the above quantities are more conveniently expressed by travelling modes, i.e. transmitted and reflected waves, both characterized by their own spatial profile and propagation constants, with specific dispersion properties. The power carried by acoustic waves is found as the transverse integral of their stress tensor and the time derivative of their mechanical displacement^26^:

3 $$P={\int }_{dS}\hat{x}\cdot ({\partial }_{t}{\bf{U}}\cdot {\bf{T}})$$

where *S* is the cross-section of the AlN membrane. In the phasor domain *ω*, the real part of *P* provides the mode power. If **U** and **T**, in equation (3), are related to different modes, the orthogonality implies zero cross power.

The (two) mechanical eigenmodes supported by the AlN membrane at 1 GHz are shown in Fig. 2b. Their relative powers, resulting from the IDT excitation, are reported in Fig. 2c: since the total mechanical power is normalized to 1 W, by using expression (3), mode powers are complementary to 1 W, as expected from ortho-normality. The space Fourier transform of the profile of the mechanical displacement along the *x* axis at fixed vertical position (*y* = 0.72 µm) provides the spectral composition of the two propagating modes, having *β*_1_ ≈ 5.8e5 rad/m @ *f* = 1 GHz and *β*_2_ ≈ 6.6e5 rad/m @ *f* = 1 GHz respectively. As reported in Fig. 2d, both of *u* and *v* displacements contribute to mode 1 (blue spectrum), whereas the predominant contribution of mode 2 (red spectrum) mainly comes from the *v* component.

### Multiphysic analysis of surface acoustic waves in periodic waveguides excited by IDT

**Figure 3:**
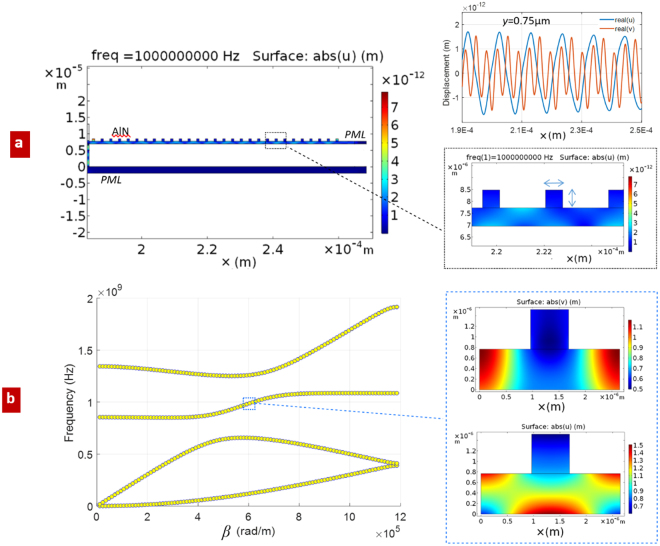
Simulation of excited mechanical waves in the suspended periodic AlN membrane; (**a**) calculated *u* component of the displacement, and (*u*, *v*) profiles along a cut line in *x* direction, at fixed vertical position in the membrane *y* = 0.72 µm; (**b**) dispersion curves of the periodic membrane and modal solutions @ 1 GHz.

In this section, a periodically patterned AlN suspended membrane is assumed as the mechanical waveguide that follows the IDT. Such periodic structure is particularly suited to TRL calibration, as explained in Section 4, leading to the possibility to work with a single mechanical mode around the operation frequency. Simulation results are reported in Fig. 3.

A detailed example of numerical TRL calibration, starting from results of Fig. 3, will be provided in Section 2.4 and Section 3. For numerical checking, the procedure presented above will be repeated in different conditions. Further numerical validations will be provided, in Section 3, by reporting a comparison with results obtained by an independent calculation.

### Results: numerical TRL calibration to de-embed the electro-mechanical transduction

In this section, we report the simulation results coming from the Thru-Reflection-Line calibration applied to the two terminal device of Fig. 1. Theory and details of the method can be found in Section 4.

For the calibration, we assume a length *L* (see Section 4) equal to the length of the unit cell of the periodic membrane: by this choice, the corresponding induced phase delay of the mechanical mode is about a quarter of its wavelength, which is the best numerical condition for calibration purpose^27^.

**Figure 4:**
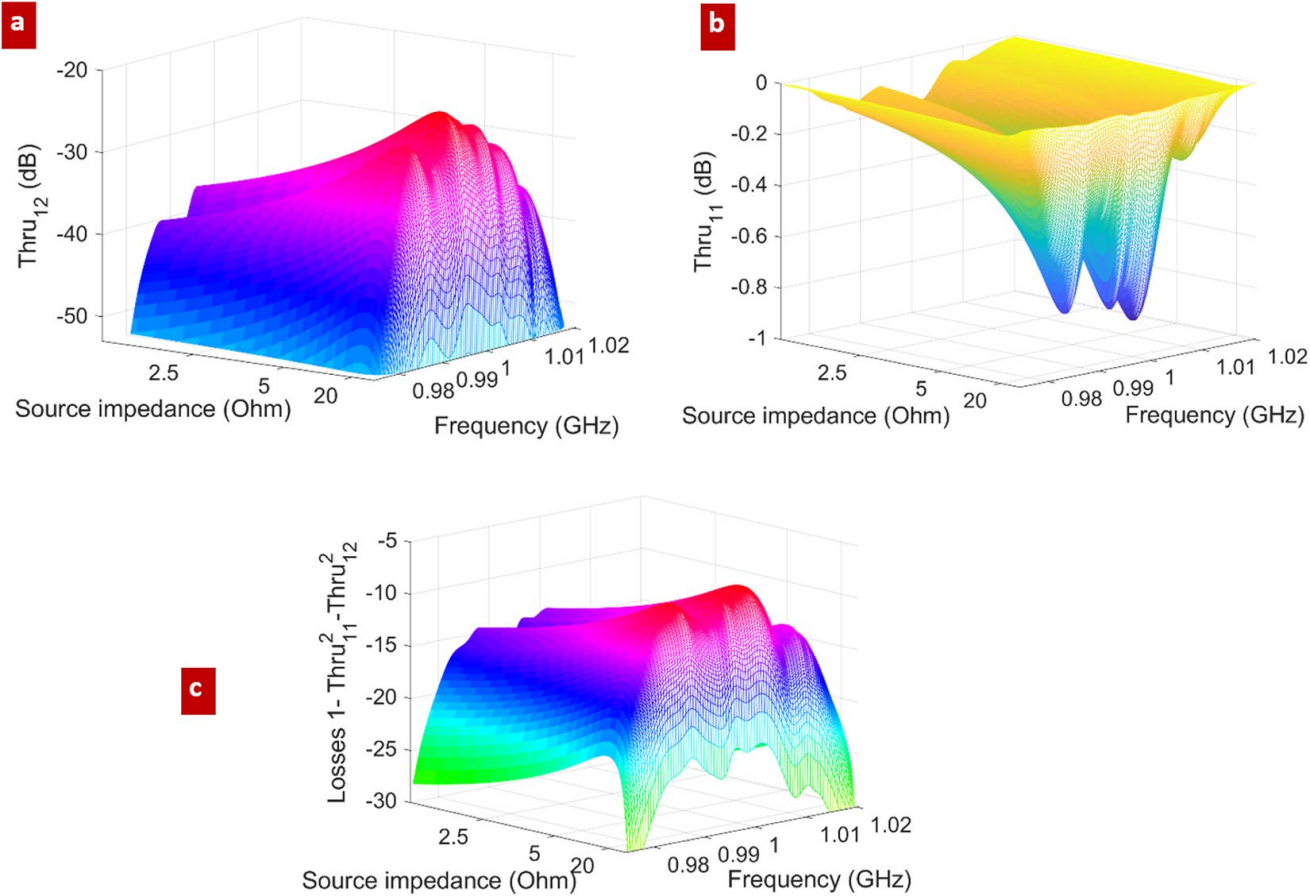
“Thru” calibration results; (**a**) simulated Thru_11_ (=Thru_22_); (**b**) simulated Thru_12_ (=Thru_21_), and (**c**) power lost in Thru configuration, as a function of the frequency and of the source impedance.

The numerical results about the Thru coefficients are shown in Fig. 4 (for space reasons, Line and Reflection coefficients are not shown). The scattering parameters are plotted as a function of frequency and source impedance: standard multiphysic simulators usually allow, among other choices, for a direct voltage excitation of the two IDT terminals, which easily leads to an impedance representation of two-port circuits. Basing on this impedance representation, different settings of the source impedance provide different values for the [Thru, Reflection, Line] scattering parameters. The source impedance, which is assumed to be the same at the two electric terminals, is set to 17.5 Ohm to minimize the back electromagnetic reflection at the IDT terminals. Accordingly, this choice provides a maximum for the transmittance Thru_12_ (Fig. 4a), which translates into having the highest coupling between the two IDT terminals in terms of electrical power.

By applying equation (6) and equation (8) of Sections 4, considering that *β* is known from previous analysis (Fig. 3b), we obtain the scattering parameters S_11_, S_12_ (equal, because of the reciprocity, to S_21_) and S_22_, of the IDT-to-waveguide transition (Fig. 1a, with *n* = 1). The subscripts 1 and 2 here refer to electrical and mechanical ports respectively.

**Figure 5:**
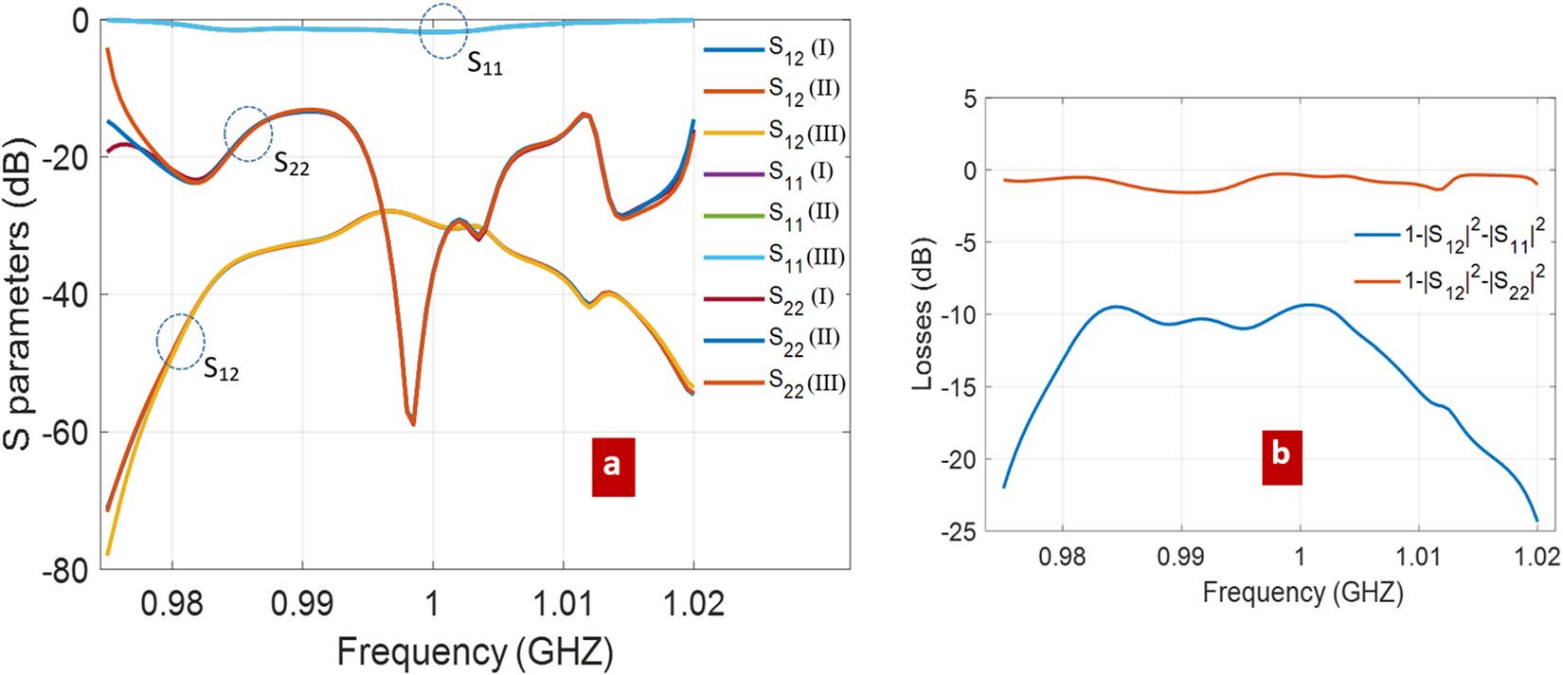
Calibrated S parameters and losses; (**a**) calibrated parameters of the embedding IDT-to-waveguide transition: S_11_, S_22_, S_12_ (= S_21_); (I) *W* = 16 unit cells and *L* = 1 unit cell, (II) *W* = 16 unit cells and *L* = 2 unit cells, (III) *W* = 17 unit cells and *L* = 1 unit cell; (**b**) net power lost in case of excitation from the mechanical waveguide (red), and from the IDT (blue): losses are due to radiation of mechanical waves into the substrate.

Results are shown in Fig. 5a: cases (I), (II), and (III) in the legend refer to different conditions for the calibration procedure: in (I) and (II) *L* = 2.65 µm and *L* = 5.3 µm respectively, with reference section (see Fig. 1b) for the mechanical port at *W* = 16 unit cells, while in (III) *L* = 2.65 µm, but the mechanical section is at *W* = 17 unit cells. It is remarked that the evanescent modes around the IDT termination are properly accounted in the simulation, i.e. closed to their matched ports, providing that the reference sections are placed at a proper distance away from the IDT termination.

The above repeated (I, II, III) simulations served to check the numerical consistency of the calibration approach. Some numerical discrepancies among them start to appear at the edges of the considered frequency band, due to the almost complete reflection S_11_ (≈0 dB), and to the almost vanishing transmission S_12_ (<−40 dB), which make the calibration potentially inaccurate. Such low transmission follows from the frequency bandgaps, that appear to be moving away from the central frequency of 1 GHz (Fig. 3b).

The power lost in the IDT-to-waveguide transition is reported in Fig. 5b. Interestingly, losses are much higher when the transition is excited from the mechanical port (red curve). In this case, the back scattering is small (S_22_), and the mechanical power is mostly lost due to transmission to substrate waves. In comparison, the high reflection experienced by the electromagnetic signal at port 1 (S_11_) implies low transmission, but also low losses (blue curve).

In close analogy with experimental TRL calibration, which is intrinsically robust against systematic errors (imperfections/connectors/etc.), Fig. 5a shows that also the numerical emulation of TRL is not too much impacted by systematic numerical errors. This is an important consequence of the fact that calibration involves simulations (Thru, Reflection, and Line) which differ from each other only in the limited region of the suspended membrane. In particular, the IDT and its underlying regions are not changed when passing from one calibration step to another.

## Independent Validation of Numerical Results and Discussion

**Figure 6:**
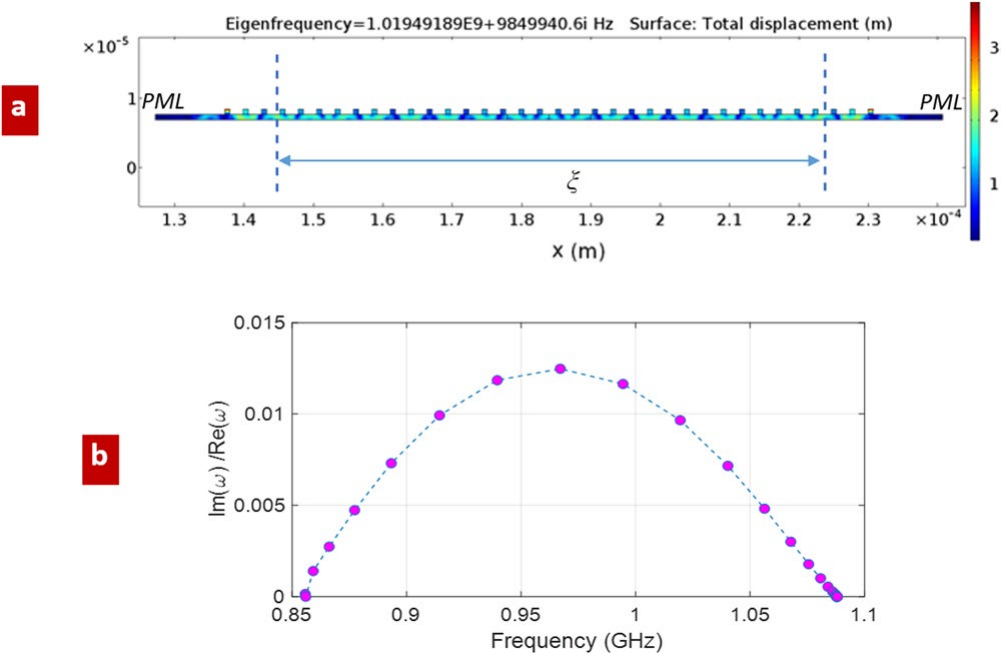
Simulation results for a mechanical cavity of length *ξ*; (**a**) total displacement at a resonant frequency of 1.019 GHz; (**b**) resonant frequencies of the cavity, calculated by FEM solver: ratios Im(*ω*)/Re(*ω*) are reported.

Let us consider a mechanical resonant cavity formed by a piece, of length *ξ*, of the AlN periodic waveguide of section 2.3 terminated on semi-infinite uniform waveguides at both sides (Fig. 6a).

In a transmission line representation, the resonance condition, with impedance load termination *z*_*l*_ at left and right sides, can be expressed as

$${z}_{l}+{z}_{0}\frac{{z}_{l}\,\cos \,\theta +j{z}_{0}\,\sin \,\theta }{{z}_{0}\,\cos \,\theta +j{z}_{l}\,\sin \,\theta }=0$$

where *z*_0_ is the characteristic impedance and θ = *β*(ω)*ξ*. By imposing an equivalent resonant condition, involving only the reflection coefficient *Γ* at the side terminations, there is no need to use the concepts of impedance load and characteristic impedance:

$$\frac{(1+{\rm{\Gamma }})}{(1-{\rm{\Gamma }})}+\frac{(1+{\rm{\Gamma }})\cos \,\theta +j(1-{\rm{\Gamma }})\sin \,\theta }{(1-{\rm{\Gamma }})\cos \,\theta +j(1+{\rm{\Gamma }})\sin \,\theta }=0$$

The solution for complex *β* = *β*′ + *jβ*″ follows as

4 $$\beta (\omega )=[arc\,\tan (j\frac{1-{{\rm{\Gamma }}}^{2}}{1+{{\rm{\Gamma }}}^{2}})+n\pi ]{\xi }^{-1}$$

where *n* is an integer number that defines the admissible solutions.

The reference sections for *Γ* (dashed vertical lines in Fig. 6a) are assumed relatively far from the left and right sides of the cavity in a way that the non-propagating mechanical modes, excited at the edge discontinuities, can be considered as numerically extinguished.

Considering that *β*(*ω*) is known from the dispersion relation of the periodic waveguide (i.e. the Floquet periodic condition for the unit cell), the complex resonant frequencies can be find by simply inverting *β*(*ω*) in equation (4). The quality factor follows as the ratio between real and imaginary parts of *ω*. Note that material-induced loss mechanisms (such as thermo-elastic bending) has been neglected for the sake of discussion. Considering a local linearization of *β*(*ω*), around *ω*_0_, the sound velocity in the corrugated structure *v*_*m*_ = 1/(∂*β*/∂*ω*) comes into play (*v*_*m*_ ≈ 5·10^3^ m/s @ 1 GHz):

5 $$\omega ={\omega }_{0}+j{v}_{m}\beta ^{\prime\prime} ({\omega }_{0})$$

As expected, the quality factor is inversely to proportional to *v*_*m*_: hence, it increases for frequencies approaching the band gaps.

The spacing between consecutive resonant frequencies is found, from equation (5), to be about 0.024 GHz at around 1 GHz, in accordance to the numerical FEM calculation of Fig. 6b.

**Figure 7:**
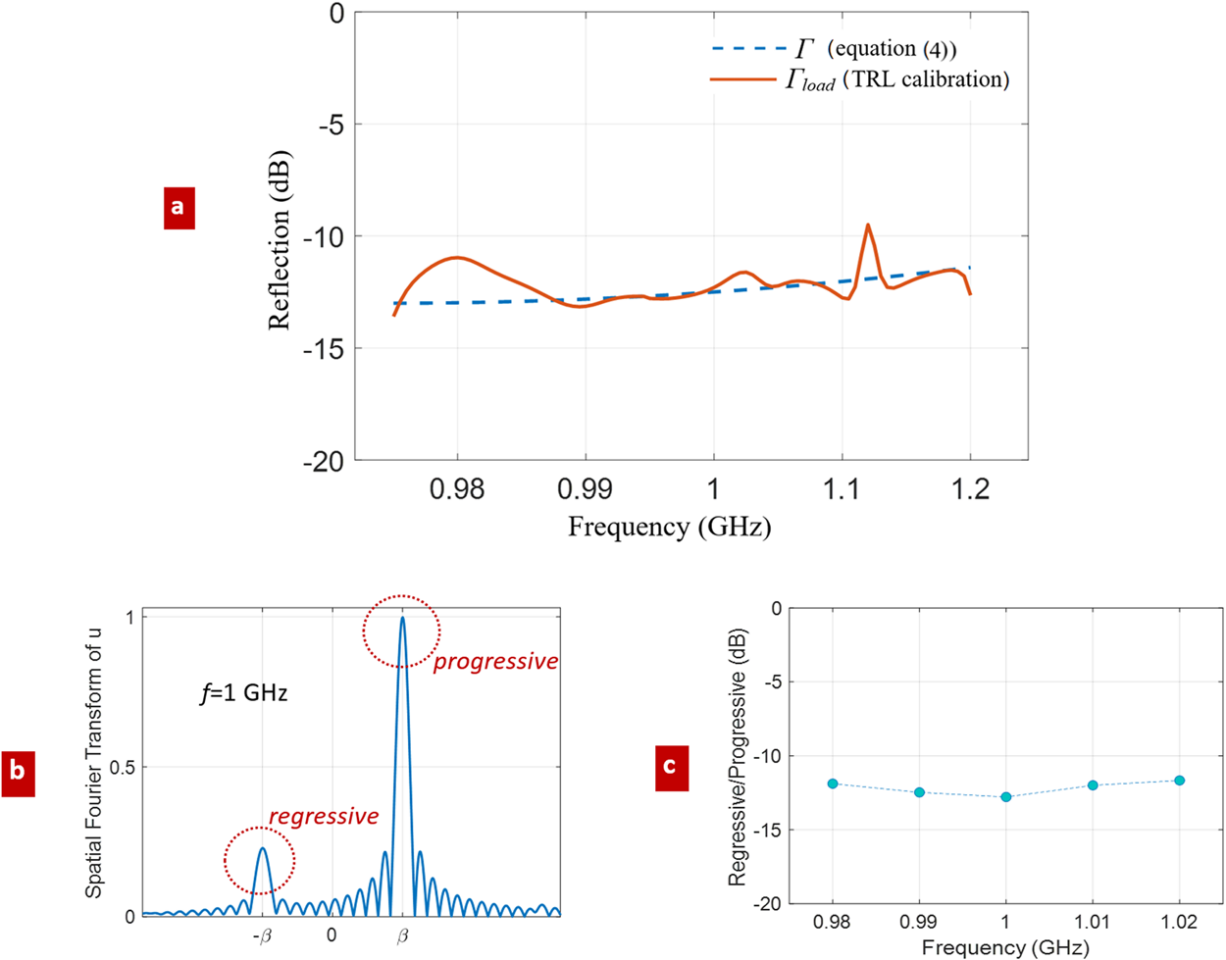
Simulations for independent validation of the TRL calibration; (**a**) reflection coefficient of the terminated periodic waveguide: comparison between the TRL-calibration *Γ*_*load*_ (red line), and *Γ* obtained combining equations (4, 5) and FEM simulation of Fig. 6b (blue dashed line); (**b**) Spatial Fourier transform of the *u* horizontal profile of Fig. 3a; (**c**) estimation of *Γ*_*load*_, by the ratio between the regressive and progressive wave amplitudes in spectral domain.

Since we know - from FEM simulation of the resonant cavity - the Im(*ω*)/Re(*ω*) ratios associated to the resonant frequencies (Fig. 6b), it is possible to find the corresponding values of *Γ*, that should to be inserted in equation (4) to match the same Im(*ω*)/Re(*ω*) ratios. These *Γ* values are plotted in Fig. 7a (the blue dashed curve “*Γ* periodic termination”). At this point, we can compare *Γ* with the reflection coefficient *Γ*_*load*_ (red curve of Fig. 7a) obtained by the de-embedding TRL procedure, i.e. equation (7) of Section 4. Although some numerical fluctuations are present, due to the large computational domain needed to include the IDT, the validity of the calibration approach is evident. To further explain such fluctuations, we must mention another limit of the simulations performed, which is actually an intrinsic limit of the TRL calibration, namely the requirement of not too small reflection from the unknown load (not perfectly well satisfied in our case) being the resulting *Γ*_*load*_ as low as about −11.5/−13 dB. The TRL approach could be systematically repeated over many equivalent setups, e.g. different values of *L*, to average results and potentially reduce the impact of numerical errors (only few different simulations are averaged to obtain the results of Fig. 7a).

For improving the numerical check, let us provide another independent (rough) estimation of *Γ*_*load*_ by the ratio between the amplitudes of regressive and progressive wave amplitudes in the periodic membrane, as follows from the evaluation of their corresponding spectral components −*β* and +*β* (Fig. 7b). The result is shown in Fig. 7c: the estimated *Γ*_*load*_ lies correctly in the range [−11.5 dB, −13 dB]. It must be underlined that this test makes sense only if S_22_ is negligibly small: in the present case, our calibration suggests, *a posteriori* (see Fig. 5a), that S_22_ is small but not actually negligible, thus explaining the little difference with the blue dashed line of Fig. 7a.

In conclusion, we have shown rigorous application of Thru-Reflection-Line (TRL) calibration aimed to characterize the coupling between electromagnetic and mechanical waves in SAW-based devices, involving large IDT structures and including a periodically patterned membrane as mechanical waveguide. To provide a numerical example, the S-parameters of a IDT-to-waveguide transition has been computed, and such calculation has been numerically verified by independent analysis.

The S-matrix characterization is potentially important for synthesis purposes, since it allows the numerical de-embedding of an acoustic device under test (DUT), acting only on the external electric terminals. Moreover, TRL calibration is quite a general approach, since it applies also to devices having nonlinear and/or active features: the only requirement is linearity of the embedding circuit (Fig. 1b).

Potential limits and challenges of the present analysis reside (i) in the high aspect ratio of typical multiphysic simulations, (ii) in the rapidly varying frequency response of large-IDT excitation, (iii) the impact of losses and mechanical coupling to the substrate, which reduces sensitivity in the calibration procedure. Ongoing work aims to apply the proposed calibration method for the study of opto-mechanical devices, phonon-photon interaction and nonlinear effects in micro- and nano-cavities.

## Methods

In this section, we report briefly about the Through Reflection Line (TRL) procedure, which is a well-established and well-known technique for experimental calibration of 2-port microwave devices^27^. The approach can be extended to multi-port circuits, characterized by many physical ports and/or by ports with many accessible modes^28–31^.

The aim of the standard TRL calibration is to extract information, namely the scattering matrix S over the frequency band of interest, about a device under test (DUT), which is not directly accessible by the available measurement tool, which is typically a vector network analyzer (VNA). In fact, a linear error box is usually interposed between the VNA and the DUT due to the presence of connectors, adapters etc. The S-matrix of this linear error box must be removed, as it causes undesired distortion of the DUT response. In the context of the present work, we refer to the error box as “embedding” circuit and to the DUT as “embedded” circuit. Looking at Fig. 1b, the embedding is clearly given by the transition from the electrical terminal to the suspended membrane, and the embedded circuit is given by a possible patterned mechanical circuit. The latter may consist of resonators, filters, metamaterials, phononic crystals, OM cavities (in this case, active elements also) and mechanical sensors.

To filter out the embedding circuit means to remove the effect of whole IDT structure together with its piezoelectric action, as well as the complicated effects of parasitic mechanical waves, mechanical radiation losses and absorption by the substrate, by the underline layers, and by any region physically connected to the IDT. Thus, the TRL calibration provides a direct way to de-embed an acoustic/mechanical circuit (DUT) *by making use only of electrical measurements from the input/output IDT terminals*. There is no need to access the internal mechanical waveguide connecting the DUT, nor to know any other parameters apart from geometry and size of the simulated structure. Clearly, this could be of great help in the design and synthesis of acoustic devices, and/or in the physical characterization of the material forming the mechanical waveguide. The transition IDT-to-mechanical waveguide is simultaneously characterized in the frequency band of interest, in terms of efficiency of the electrical-mechanical transduction and associated losses.

**Figure 8:**
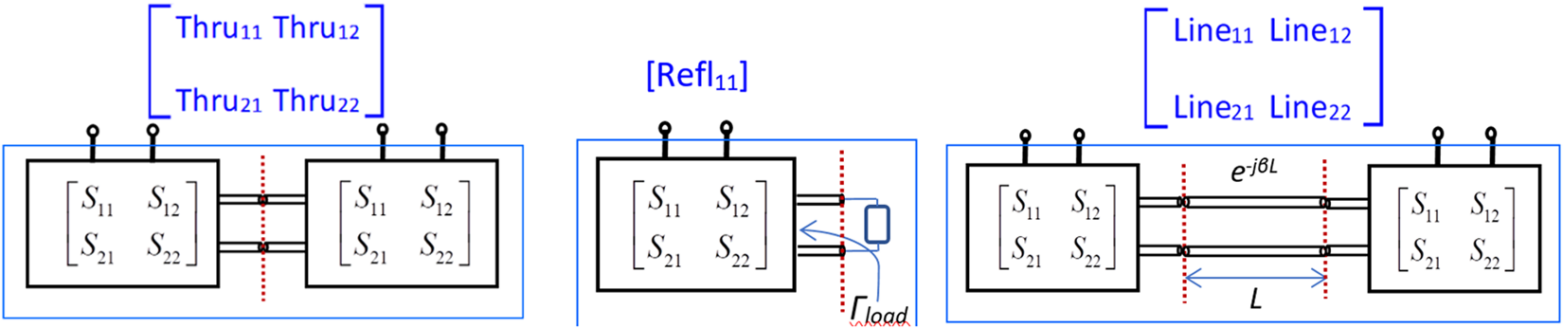
Schematic design of the Thru, Reflection, and Line simulations.

Going into detail, the TRL calibration consists of three independent measurements (here simulations), under known (but different) conditions, namely “Thru”, “Reflection”, and “Line”, as shown in Fig. 8. The embedding circuit is assumed to be reciprocal, which implies that S_21_ = S_12_.

The final goal is to find the following five circuit parameters: the scattering coefficients of the embedding circuit (S_11_, S_12_, S_22_), the propagation constant of the mechanical transmission line (*β*) of the “Line” simulation and the mechanical reflection from the unknown load (*Γ*_*load*_) in the “Reflect” simulation. The following assumptions are made: (i) mono-modal mechanical waveguide, (ii) knowledge of all geometrical parameters, with a special attention to the transmission line length *L* in the “Line” simulation and (iii) perfect symmetry of the whole computational domain, which is guaranteed by “mirroring” the computational mesh.

Basing on these assumptions, TLR simulations provide the five equations needed to solve for the five unknown complex parameters in terms of known ones (Thru_11_, Thru_12_, Refl_11_, Line_11_, Line_12_):

6 $${{\rm{Thru}}}_{11}={S}_{11}+\frac{{S}_{22}{S}_{12}^{2}}{1-{S}_{22}^{2}}={{\rm{Thru}}}_{22}\,{{\rm{Thru}}}_{12}={S}_{11}+\frac{{S}_{12}^{2}}{1-{S}_{22}^{2}}={{\rm{Thru}}}_{21}$$

7 $${{\rm{Refl}}}_{11}={S}_{11}+\frac{{S}_{12}^{2}{{\rm{\Gamma }}}_{load}}{1-{S}_{22}{{\rm{\Gamma }}}_{load}}={{\rm{Refl}}}_{22}$$

8 $${{\rm{Line}}}_{11}={S}_{11}+\frac{{S}_{22}{S}_{12}^{2}{e}^{-2\beta L}}{1-{S}_{22}^{2}{e}^{-2\beta L}}={{\rm{Line}}}_{22}\,{{\rm{Line}}}_{12}={S}_{11}+\frac{{S}_{12}^{2}{e}^{-\beta L}}{1-{S}_{22}^{2}{e}^{-2\beta L}}={{\rm{Line}}}_{21}$$

Obviously if one or more, among the five targeted parameters, is actually known in advance, the above system of equations can be simplified accordingly. For instance, if the propagation constant *β* is known, as it was assumed in Section 3, equation (7) can be solved separately from the others.

In the present work, we have considered periodically patterned suspended membrane as mechanical waveguide, namely the waveguide studied in Section 2.3. This may seem to complicate the analysis, but it actually makes the calibration possible in the form of equations (6–8) since the periodic waveguide is designed to support only one propagating mode. Otherwise, with two or more propagating modes, an extended calibration procedure is needed, to combine more than three independent simulations, in order to find an increased number of unknown parameters. The assumption of a periodic structure also allows to better fit practical situations where the mechanical waveguides are constituted by phononic crystals/metamaterials and nano/micro patterned beams or layers.

### Data availability

The datasets generated during and/or analysed during the current study are available from the corresponding Author on reasonable request.
